# Assessing the Pathogenicity of In-Frame *CACNA1F* Indel Variants Using Structural Modeling

**DOI:** 10.1016/j.jmoldx.2022.09.005

**Published:** 2022-10-01

**Authors:** Shalaw R. Sallah, Panagiotis I. Sergouniotis, Claire Hardcastle, Simon Ramsden, Andrew J. Lotery, Nick Lench, Simon C. Lovell, Graeme C.M. Black

**Affiliations:** ∗ Division of Evolution and Genomic Sciences, School of Biological Sciences, Faculty of Biology, Medicines and Health, University of Manchester, Manchester Academic Health Science Centre, Manchester, United Kingdom; † Manchester Centre for Genomic Medicine, Manchester University NHS Foundation Trust, Manchester Academic Health Science Centre, St. Mary’s Hospital, Manchester, United Kingdom; ‡ Faculty of Medicine, University of Southampton, Southampton, United Kingdom; § Congenica Ltd., BioData Innovation Centre, Wellcome Genome Campus, Hinxton, Cambridge, United Kingdom

## Abstract

Small in-frame insertion-deletion (indel) variants are a common form of genomic variation whose impact on rare disease phenotypes has been understudied. The prediction of the pathogenicity of such variants remains challenging. X-linked incomplete congenital stationary night blindness type 2 (CSNB2) is a nonprogressive, inherited retinal disorder caused by variants in *CACNA1F*, encoding the Ca_v_1.4α1 channel protein. Here, structural analysis was used through homology modeling to interpret 10 disease-correlated and 10 putatively benign *CACNA1F* in-frame indel variants. CSNB2-correlated changes were found to be more highly conserved compared with putative benign variants. Notably, all 10 disease-correlated variants but none of the benign changes were within modeled regions of the protein. Structural analysis revealed that disease-correlated variants are predicted to destabilize the structure and function of the Ca_v_1.4α1 channel protein. Overall, the use of structural information to interpret the consequences of in-frame indel variants provides an important adjunct that can improve the diagnosis for individuals with CSNB2.

Congenital stationary night blindness type 2 (CSNB2), also known as incomplete CSNB or congenital cone-rod synaptic disorder, is a nonprogressive, childhood-onset inherited retinal disorder. It is inherited as an X-linked trait and results in myopia, nystagmus, reduced visual acuity, and, occasionally, night vision problems and/or light hypersensitivity in affected individuals. Genetic testing together with clinical evaluation are essential for the diagnosis of CSNB2,^1^^,^^2^ which is mainly caused by pathogenic variants in the *CACNA1F* gene, encoding the Ca_v_1.4α1 transmembrane calcium ion channel.^3^ Ca_v_1.4α1 sustains continuous calcium-dependent glutamate release from the photoreceptors to bipolar cells in the retina.^4^ More than 200 CSNB2-correlated *CACNA1F* variants have been reported to date, with the majority being missense (*n* = 96) or small insertion-deletion (indel; *n* = 67) variants.

Indel variants are the second most common type of genetic variation after single nucleotide changes,^5^^,^^6^ and they can occasionally have a drastic effect on protein structure, leading to destabilization and misfolding.^7^^,^^8^ A number of computational tools have been developed for the assessment of these changes, including SIFT-indel (Sorting Intolerant From Tolerant),^9^ CADD (Combined Annotation-Dependent Depletion),^10^ MutationTaster2,^11^ PROVEAN (Protein Variation Effect Analyzer),^12^ DDIG-in (Detecting DIsease-causing Genetic variations due to indels),^13^ VEST-indel (Variant Effect Scoring Tool),^14^ and MutPred-indel.^15^ Although high prediction performance has been reported for some of these tools, with an area under the receiver-operating characteristic curve of up to 0.91,^15^ their performance in clinical diagnostic settings is often suboptimal, and clinical interpretation of these variants remains challenging.

Previously, clinical data were combined with homology modeling to interpret *CACNA1F* missense variants with high accuracy.^16^ Here, a similar approach based on protein structure analysis was used to distinguish disease-correlated from benign small in-frame *CACNA1F* indels.

## Materials and Methods

### Variant Data Sets

In-frame deletions, insertions, and indel variants in *CACNA1F* involving <51 nucleotides were retrieved (Ensembl, *https://useast.ensembl.org/index.html*, accession number ENST00000376265.2) for structural analysis. The Human Gene Mutation database Professional version (QIAGEN, Cardiff, UK)^17^ was used to obtain changes correlated with clinical phenotypes (ie, disease correlated). The ClinVar^18^ database was also used to retrieve variants, including disease-correlated and putatively benign changes (*https://www.ncbi.nlm.nih.gov/clinvar*). A biomedical literature search was conducted at the same time using the search term “CACNA1F AND mutation∗” to expand the list of variants. The Genome Aggregation Database (gnomAD) version 2.1^19^ was also interrogated at the same time to retrieve presumably benign variants reported in male subjects (gnomAD, *http://gnomad.broadinstitute.org*, last accessed April 25, 2020). In addition, the in-house Manchester Genomic Diagnostic Laboratory (MGDL) database, a resource including clinical genetic test results from individuals tested at a United Kingdom–accredited genomic laboratory (Clinical Pathology Accredited identifier no. 4015), was interrogated for both disease-correlated and putatively benign in-frame indels (accessed April 30, 2020). The American College of Medical Genetics and Genomics and the Association of Molecular Pathology (ACMG) guidelines^20^ were used to evaluate the variants.

### Clinical Evaluation of Affected Individuals

Clinical assessment was performed in selected study subjects who carried *CACNA1F* in-frame indel variants and presented to the tertiary ophthalmic genetics service at Manchester University NHS Foundation Trust (Manchester, UK) or University Hospital Southampton NHS Foundation Trust (Southampton, UK). This assessment included visual acuity testing, dilated fundus examination, digital widefield fundus imaging, fundus autofluorescence imaging, and optical coherence tomography (OCT). The Optos system (Optos PLC, Dunfermline, Scotland, UK) was used to obtain widefield images, and the Topcon DRI OCT Triton device [Topcon (Great Britain) Medical Limited, Newbury, Berkshire, UK] was used to obtain OCT scans. Visual electrodiagnostic testing was performed in most cases; the protocols used incorporated the standards of the International Society for Electrophysiology of Vision for full-field electroretinography.^21^

### Homology Modeling

Because an experimentally determined three-dimensional Ca_v_1.4α1 structure was unavailable, a homology model was generated by using MODELLER version 9.17,^22^ which uses satisfaction of spatial restraint techniques to generate the model coordinates. The model was used to assess the pathogenicity of all variants. The *CACNA1F* sequence from UniProt^23^ (UniProt identifier O60840) was used, and the rabbit Ca_v_1.1 complex from the Protein Data Bank^24^ was identified as the most appropriate template structure (Protein Data Bank identifier 5GJV); Clustal Omega version 1.2.4 with default parameters^25^ was used to align the relevant sequences. The generated Ca_v_1.4α1 model with the lowest Discrete Optimized Protein Energy score was selected of a total of five. The MolProbity score, which is a combination of the clash score, rotamer, and Ramachandran evaluations, was used to evaluate the quality of the model.^26^ PyMOL (The PyMOL Molecular Graphics System version 1.8; Schrödinger, LLC, New York, NY) was used to visualize the model. The possible loss of hydrogen bonding interactions, which were identified by using MolProbity,^27^ were considered destabilizing to the protein structure. The possible disruptions to hydrophobic or ionic interactions, identified through visual inspection of the variants, were deemed to contribute to protein instability. All features were combined to make an overall prediction.

### Sequence Conservation Analysis

A multiple sequence alignment of the 11 *CACNA1F* orthologues available in the commercial software Alamut Visual, version 2.11 (Interactive Biosoftware, Rouen, France) was performed by using MUSCLE^28^ to measure conservation. A conservation score between 1 and 9 for the *CACNA1F* sequence residues was calculated by generating a profile in which the alignment was converted into a position-specific scoring system.^29^ The frequency of the residues at each position was scored and used to measure conservation by employing the substitution matrix BLOSUM62.^30^

## Results

### *CACNA1F* In-Frame Indel Variant Identification

First, in-frame *CACNA1F* indel variants identified in individuals with CSNB2 were collected. Ten such variants were found, including seven from the Human Gene Mutation database, one from the biomedical literature,^31^ and two previously unreported variants from the MGDL database (submitted to ClinVar^18^); these variants collectively formed data set A (Table 1).

**Table 1:** *CACNA1F* Variants (Ensembl, *https://useast.ensembl.org/index.html*, accession number ENST00000376265.2) Involving In-Frame Insertion and/or Deletion <51 Nucleotides Identified in Individuals with CSNB2 (Data Set A) or in Individuals Who Either Did Not Have CSNB2 or Who has CSNB2 but had a Previously Identified Pathogenic Variant (Data Set B)

Variant	Data set	Is residue modeled?	Conservation	Source
*c.466-469delAGCGinsGTAGGGGTGCTCCACCCCGTAGGGGAGCTCCACC p.(Ser156_Ala157delins ValGlyValLeuHisProValGlyValLeuHisPro)*	A	Yes	3, 2	32
*c.495_496insTACCTA p.(Leu165_Leu166insTyrLeu)*	A	Yes	–	33
*c.952_954delTTC p.(Phe318del)*	A	Yes	3	32
*c.1004_1009delTGCTCT p.(Val335_Tyr337delinsAsp)*	A	Yes	1, 1, 1	3
*c.1612_1632delCTCGTCTTCCTCAACACGTTG p.(Leu538_Leu544del)*	A	Yes	1, 1, 2, 1, 1, 1, 6	MGDL
*c.2829_2830delGGinsCT p.(Leu943_Asp944delinsPheTyr)*	A	Yes	1, 1	3
*c.3009_3011delCAT p.(Ile1003del)*	A	Yes	1	32
*c.3658_3669delGTCCATGGCATA p.(Tyr1220_Asp1223del)*	A	Yes	8, 7, 3, 3	31
*c.3691_3702delAGTGAAGAGGCC p.(Gly1231_Thr1234del)*	A	Yes	7, 3, 1, 5	34
*c.4093_4095delAAC p.(Asn1365del)*	A	Yes	1	MGDL
*c.1466_1468delAGG p.(Glu489del)*	B	No	8	19
*c.5195_5197delAAG p.(Glu1732del)*	B	No	8	19
*c.5866_5868delGAG p.(Glu1956del)*	B	No	8	19
*c.2442_2444delGGA p.(Glu825del)∗*	B	No	3	MGDL
*c.2457_2474dupAGAGGAAGAAGAGGAAGA p.(Glu820_Glu825dup)∗*	B	No	–	19
*c.2466_2474delAGAGGAAGA p.(Glu823_Glu825del)∗*	B	No	6, 9, 9	19
*c.2445_2450delAGAAGA p.(Glu824_Glu825del)∗*	B	No	9, 9	19
*c.2466_2474dupAGAGGAAGA p.(Glu823_Glu825dup)∗*	B	No	–	19
*c.2439_2444dupGGAGGA p.(Glu824_Glu825dup)∗*	B	No	–	19
*c.2442_2444dupGGA p.(Glu825dup)∗*	B	No	–	19, MGDL

Modeled: the variants found on the modeled regions shared between the protein sequence and the template structure. The conservation scores of the deleted residues range between 1 and 9, with 1 being highly conserved and 9 being highly variable. The variants denoted with an asterisk were found in a low complexity region.

CSNB2, congenital stationary night blindness type 2; MGDL, the in-house Manchester Genomic Diagnostic Laboratory.

Next, *CACNA1F* indel variants detected in individuals who did not have CSNB2 or had CSNB2 but already carried another previously reported pathogenic variant were identified. Ten such variants were identified, including nine from gnomAD and two from MGDL (one of which was also present in gnomAD); these variants collectively formed data set B (Table 1). The variants from both data sets were next classified by a trained clinical scientist using the ACMG guidelines; the criteria used are described in Supplemental Table S1.^3^^,^^31^, ^32^, ^33^, ^35^, ^36^ From data set A, only one in 10 was defined as likely pathogenic while the remaining nine were classified as variants of unknown significance. From data set B, all variants were classified as variants of unknown significance. It was not intended to assign pathogenicity to either data set, although the two lists were considered to represent cohorts that were skewed toward carrying disease-correlated (data set A) and putatively benign (data set B) variants, respectively.

### Clinical Findings in Individuals from MGDL with *CACNA1F* In-Frame Indels

**Figure 1:**
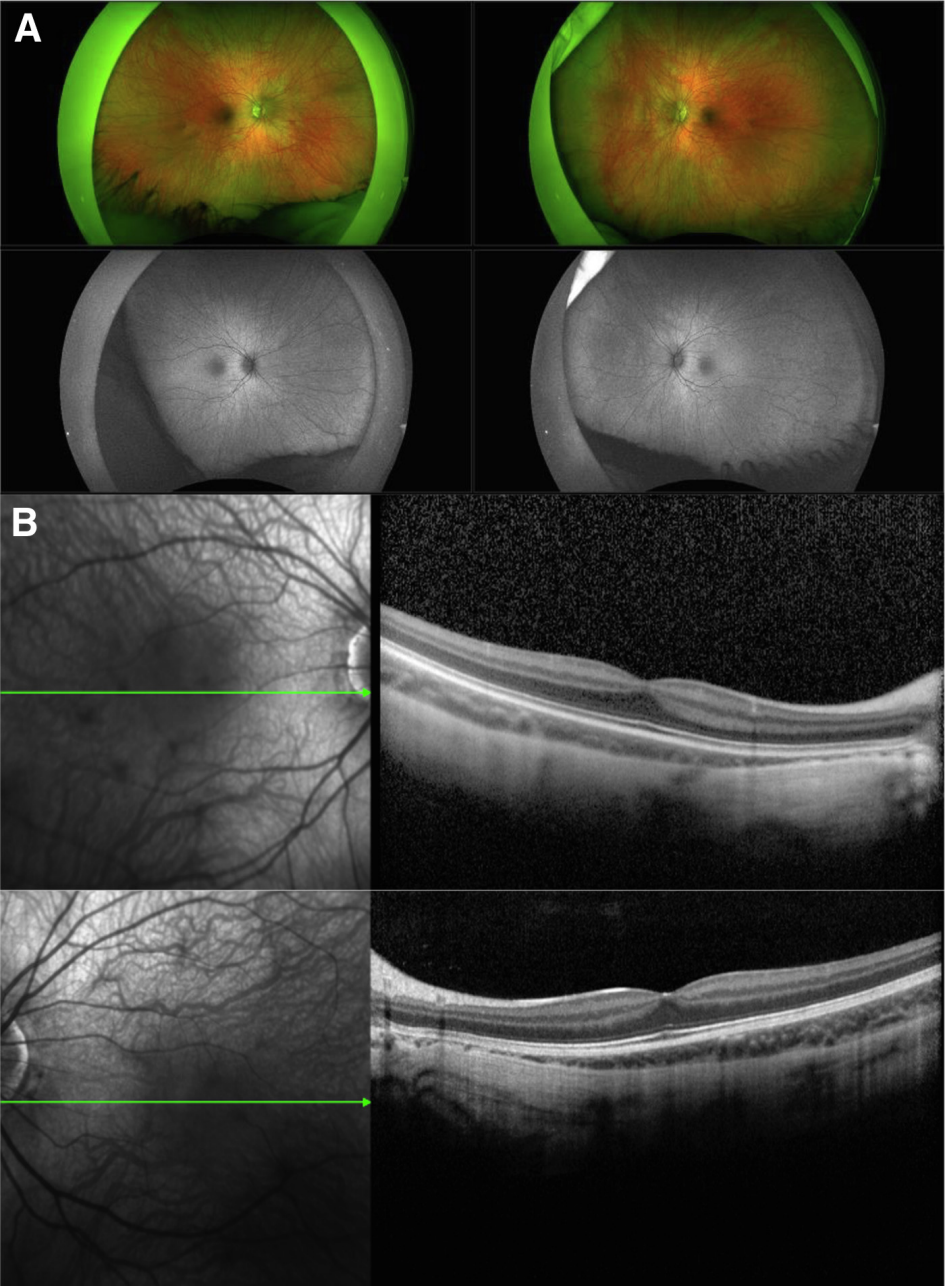
Retinal imaging of a patient with congenital stationary night blindness type 2 (CSNB2) carrying *c.4093_4095delAAC p.(Asn1365del)*. Normal widefield images of the fundus with green light autofluorescence of patient (**A**) and normal optical coherence tomography images using the Spectralis system (Heidelberg Engineering Inc., Franklin, MA) (**B**).
3 *c.2442 2444deIGGA p.(Glu825del).* This variant was identified on a single occasion when it was seen together with a known and recurrent pathogenic missense *CACNA1F* variant, *c.868C>T p.(Arg290Cys)*, in an 18-year–old man referred with a rod cone dystrophy. This novel variant was not seen in public databases.
4 *c.2442_2444dupGGA p.(Glu825dup).* This variant has not been reported in the biomedical literature or in the public databases but was identified eight times in male and female subjects within the MGDL database. In six of these eight patients, this variant was found alongside another pathogenic/likely pathogenic variant (Supplemental Table S2): two patients with rod-cone dystrophy (who were heterozygous and homozygous for mutations in *RHO* and *PDE6B*, respectively), one patient with early childhood–onset retinal dystrophy (who was homozygous for a mutation in *WDR19*), one patient with fundus albipunctatus (who was homozygous for a mutation in *RLBP1*), and two patients with Usher syndrome (who were both compound heterozygous, one for a variant in *USH2A* and the other for a variant in *CDH23*). No definitive molecular diagnosis was identified in two additional patients, one with cone dystrophy (no variants detected other than the *CACNA1F* indel change) and one with rod-cone dystrophy (who was also homozygous for a variant of unknown significance in *EYS*). This variant was seen in gnomAD with an allele frequency of 4%.

The following four *CACNA1F* in-frame variants were identified in the MGDL database and were then classified by using the ACMG guidelines:

1 *c.1612_1632delCTCGTCTTCCTCAACACGTTG p.(Leu538_Leu544del).* This previously unreported change that has not been encountered in public databases was identified in a 32-year–old man with a history of congenital nystagmus. His visual acuity was 1.3 logMAR in the right eye and 1.56 logMAR in the left eye, and he was registered as severely sight-impaired. Results of both OCT and fundus autofluorescence imaging were unremarkable. On electroretinography, the dark-adapted (rod-mediated) and light-adapted (cone-mediated) responses were significantly reduced and in keeping with the characteristic electrophysiological phenotype of CSNB2.
2 *c.4093_4095delAAC p.(Asn1365del).* This previously unreported variant that has not been encountered in public databases was identified in an 8-year–old boy with congenital nystagmus and reduced visual acuity. Results of OCT and fundus autofluorescence imaging were within normal limits (Figure 1), and the full-field electroretinograms were attenuated in the light-adapted state. Rod-mediated responses were reduced with an electronegative response strongly suggestive of CSNB2.

### Analysis of *CACNA1F* Indel Variants Using Homology Modeling

**Figure 2:**
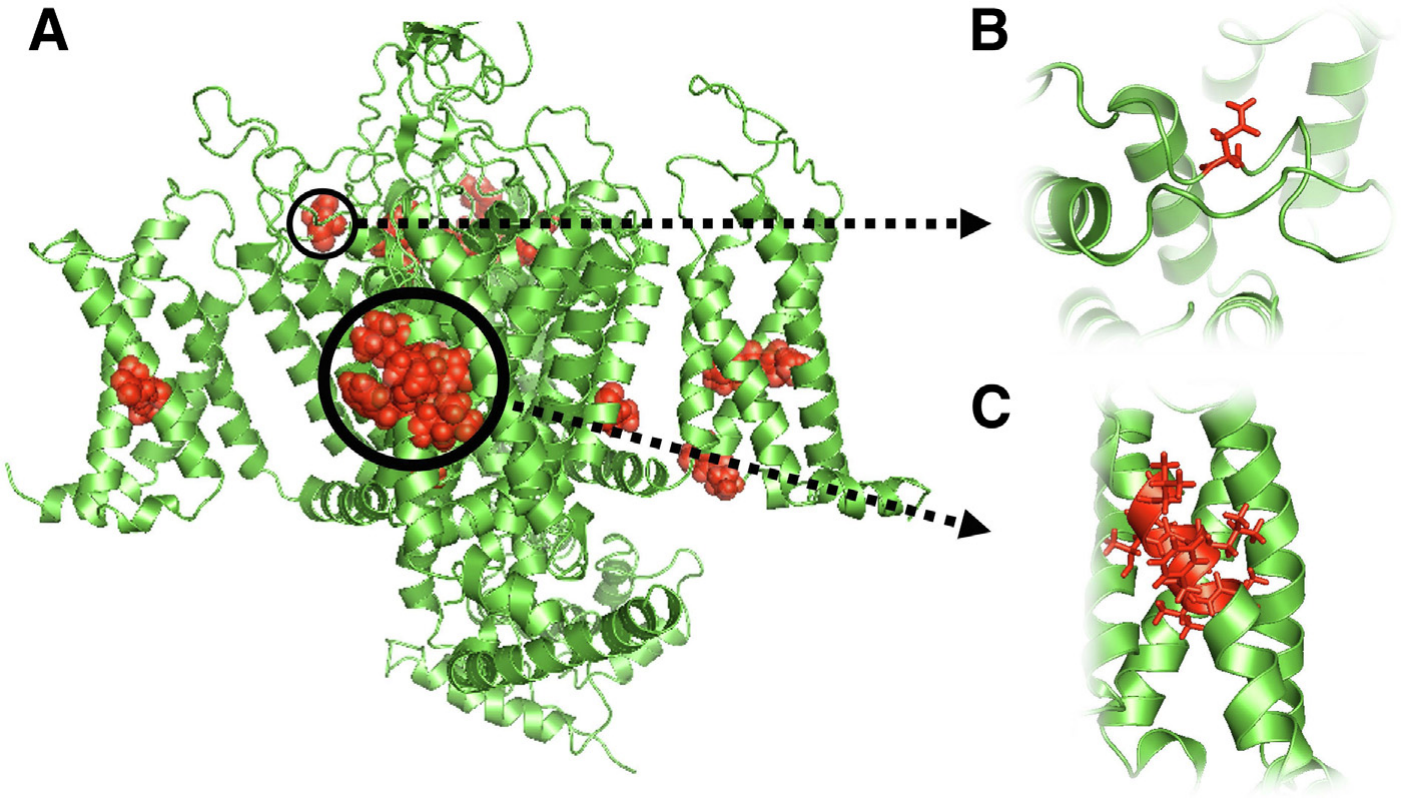
A three-dimensional model of Ca_v_1.4α1 showing the disease-correlated variants (highlighted in red) to be on the regions shared by both the template structure and the model (**A**) compared with the benign variants, none of which is on these regions. **B:** The *c.4093_4095delAAC p.(Asn1365del)* variant in a loop in the extracellular region. **C:** The *c.1612_1632delCTCGTCTTCCTCAACACGTTG p.(Leu538_Leu544del)* variant on an α-helix in a voltage-sensing domain in the transmembrane region.

A homology model of Ca_v_1.4α1 (Ensembl, *https://useast.ensembl.org/index.html*, accession number ENST00000376265.2) with a 64% sequence identity in the modeled regions (ie, the regions shared by both the model and the template structure) was generated suggesting a high similarity in structure. Model evaluation resulted in a MolProbity score of 3.33. The Ramachandran analysis of the model is presented in Supplemental Figure S1. This model was used to analyze the structural properties of all variants. Approximately two-thirds of the human Ca_v_1.4α1 protein could be modeled; this corresponds to the residues 67 to 414, 516 to 766, and 858 to 1580. Although not necessarily missing from the template protein due to missing electron density when the structure was solved, the *CACNA1F* sequence residues 1 to 66, 415 to 515, 767 to 857, and 1581 to 1977 had no reliable homologous template and could not be modeled; the template–target alignment is given in Supplemental Table S3. The sequences with no homologous template were also found to contain most of the regions that are predicted to be disordered according to IUPred^37^; the sequence prediction is provided in Supplemental Figure S2. All of the data set A variants were in regions that could be structurally analyzed (Figure 2A). In contrast, the data set B variants were all found to be in regions that could not be reliably modeled. Furthermore, all the residues altered by variants in data set B were glutamates. Seven of these 10 variants (four duplications and three deletions) were in a glutamate-rich region (including 16 residues of consecutive glutamates) that was flagged as a low complexity region and predicted to be disordered. The remaining three variants, *c.1466_1468delAGG p.(Glu489del)*, *c.5195_5197delAAG p.(Glu1732del)*, and *c.5866_5868delGAG p.(Glu1956del)*, were rare changes seen only once in gnomAD.

### Variant Conservation and Structural Analysis

**Figure 3:**
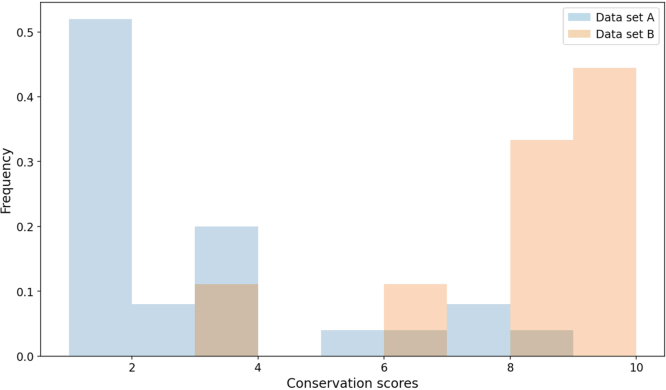
Conservation of the 25 and nine deleted residues in the nine variants found in patients with CSNB2 (data set A) and in the six variants found in healthy individuals (data set B), respectively. A low conservation score of 1 represents a highly conserved residue and a high conservation score represents a highly variable residue.

All *CACNA1F* variants in data sets A and B were then grouped into three categories depending on the degree of conservation of the affected residues: highly conserved (with a conservation score of 1 to 3), moderately conserved (with a conservation score of 4 to 6), and highly variable (with a conservation score of 7 to 9). The residues altered by variants in data set A were significantly more conserved compared with those altered by data set B variants (*U* test, *P* = 3 × 10^−5^) (Figure 3). Furthermore, all in-frame indels from data set A involved at least one residue that was highly conserved (ie, had a conservation score of 1 to 3). This was in contrast to the variants in data set B, the majority of which involved glutamates that were more likely to be variable (Table 1).

The structural impact on the protein was predicted by using the homology model for all eligible variants (ie, all variants in data set A). The two previously unreported changes are discussed in the following text; the findings for the remainder are described in Table 2, and a detailed assessment is provided in Supplemental Table S4. Overall, these analyses show that all of these variants were predicted to be destabilizing, with projected disruptions to hydrophobic interactions and/or hydrogen bonding. Nine of 10 changes were found to disrupt the structural integrity of α-helices.

**Table 2:** Structural Analysis of Small In-Frame *CACNA1F* Insertion and/or Deletion Variants Involving <51 Nucleotides Identified in Patients with CSNB2

Variant	ACMG class	Secondary structure	Hydrophobic interactions disrupted	Hydrogen bonding disrupted	Ionic interaction involved
c.466-469delAGCGinsGTAGGGGTGCTCCACCCCGTAGGGGAGCTCCACC p.(Ser156_Ala157delins ValGlyValLeuHisProValGlyValLeuHisPro)	LP	α-Helix	Possibly	Unlikely	None
c.495_496insTACCTA p.(Leu165_Leu166insTyrLeu)	VUS	α-Helix	Likely	Unlikely	None
c.952_954delTTC p.(Phe318del)	VUS	α-Helix	Possibly	Likely	None
c.1004_1009delTGCTCT p.(Val335_Tyr337delinsAsp)	VUS	α-Helix	Likely	Unlikely	Possibly (created)
c.1612_1632delCTCGTCTTCCTCAACACGTTG p.(Leu538_Leu544del)	VUS	α-Helix	Likely	Likely	None
c.2829_2830delGGinsCT p.(Leu943_Asp944delinsPheTyr)	VUS	α-Helix	Possibly	Unlikely	Possibly (lost)
c.3009_3011delCAT p.(Ile1003del)	VUS	α-Helix	Likely	Unlikely	None
c.3658_3669delGTCCATGGCATA p.(Tyr1220_Asp1223del)	VUS	α-Helix	Possibly	Likely	Possibly (lost)
c.3691_3702delAGTGAAGAGGCC p.(Gly1231_Thr1234del)	VUS	α-Helix	Likely	Likely	None
c.4093_4095delAAC p.(Asn1365del)	VUS	Loop	Unlikely	Likely	None

All of these variants were predicted to have a destabilizing effect on protein structure. The analyses were performed by using a homology model of Ca_v_1.4α1 (Ensembl, *https://useast.ensembl.org/index.html*, accession number ENST00000376265.2). Involvement of residues in hydrogen bonding was predicted using MolProbity analyses.^27^ Involvement of residues in ionic or hydrophobic interactions were identified by visual inspection of the homology model (the possibility of bond-disruption/involvement in order of likelihood: likely, possibly, and unlikely).

ACMG, the American College of Medical Genetics and Genomics and Association of Molecular Pathology guidelines^20^; CSNB2, congenital stationary night blindness type 2; LP, likely pathogenic; VUS, variant of unknown significance.

The novel variant *c.4093_4095delAAC p.(Asn1365del)* alters a loop structure, in contrast to all other variants in data set A, which affect α-helices. This loop is in a region close to the ion intake site in the extracellular region. The deletion of the Asn1367 residue is predicted to remove the hydrogen bond that it forms with the Gln1371 residue, which is likely to destabilize the interactions within the loop and the protein, affecting ion intake (Figure 2B).

The other previously unreported variant, *c.1612_1632delCTCGTCTTCCTCAACACGTTG p.(Leu538_Leu544del)*, results in the deletion of seven residues in the middle of a transmembrane helix. This leads to the disturbance of existing hydrophobic interactions between the adjacent helices. In addition, it results in a shorter helix that is likely to alter the function of the voltage-gated domain. This change is therefore highly likely to affect the structure and function of the protein (Figure 2C).

## Discussion

The current study shows that the homology model of Ca_v_1.4α1 can be used to facilitate the accurate differentiation between disease-correlated and likely benign *CACNA1F* in-frame indel variants. Diagnostic variant classification using the ACMG guidelines showed that nine of the 10 disease-correlated indels were classified as variants of unknown significance. The proposed structural modeling approach can help resolve this uncertainty and allows effective functional prediction of variant impact.

Structural analysis can be used to provide atomic-level information about the molecular mechanisms through which specific genetic variants cause phenotypic abnormalities.^38^^,^^39^ Such analyses involve two key steps: predicting three-dimensional protein structure and modeling the effect of specific sequence alterations on these complex structures. This study focused on the CACNA1F molecule and on the in-frame indel class of genetic variation. The findings suggest that these changes are more likely to be correlated with disease when altering conserved sites, including residues in α-helices (rather than loops) and/or positions outside low complexity regions; these observations are in keeping with previous studies of small indels.^9^^,^^13^^,^^15^^,^^40^^,^^41^ Notably, all 10 disease-correlated variants in this study were found to be on the modeled regions of the Ca_v_1.4α1 protein model, whereas putatively benign variants were all found on regions that lacked a homologous template structure. These unmodeled regions are poorly conserved across 10 human paralogues^42^ and correspond to regions that are predicted to be disordered.^37^ Disordered regions are flexible and lack a stable tertiary structure; they are likely to be interaction sites. Furthermore, the three-dimensional structure can be used to identify protein regions correlated with disease through variant clustering (Figure 2). As such, data suggesting alteration of structure could be used as supporting evidence in classifying variants.

The current study has notable limitations. According to the gnomAD curation team, every effort was made to exclude individuals with severe pediatric diseases from the data set^19^ (gnomAD, *http://gnomad.broadinstitute.org/faq*, accessed April 25, 2020). Although it would not be anticipated that any of these variants are correlated with ocular phenotypes, it is not possible to confirm this. Clearly, as more variants are being reported, repeating this study using a larger data set will further validate the predictive power of protein structure analysis. Using more comprehensive databases such as the UK 100,000 Genomes Project and UK Biobank, which provide genetic and clinical data, can facilitate more precise analyses and a more accurate model. Lack of a model for the regions hosting the benign variants prevented the structural assessment and the functional prediction of these variants. However, this feature of the Ca_v_1.4α1 model was also its strength as it was used in distinguishing the putatively benign variants on the unmodeled regions of the protein.^16^ Furthermore, using a homology model in the absence of an experimentally solved structure could limit the accuracy of variant assessment: the MolProbity score (structure evaluation) put the template structure (Protein Data Bank identifier 5GJV) in the 92th percentile, whereas the MODELLER-generated homology model was in the 14th percentile (100th and 0th percentiles being the best and worst, respectively). However, the MolProbity score for a homology model (using the same template structure) generated through SWISS-MODEL (a fully automated protein structure homology-modeling server that applies Hidden Markov model methods)^43^ put the homology model in the 81st percentile. More recent advances in computational protein structure prediction (eg, AlphaFold,^44^ RoseTTAFold^45^) can highly improve the modeling framework by potentially providing highly accurate three-dimensional models that are comparable to experimental structure.

Currently available tools for missense variant interpretation include Missense3D,^46^ which predicts variant’s structural impact (ie, steric clashes, provides information on other structural features). However, these tools are lacking for indel variants due to complexity in indel variant interpretation, thus in turn complicating the design of an automated variant interpretation tool. Nevertheless, automated protein modeling tools such as PhyreRisk (designed for missense variant interpretation^47^) and SWISS-MODEL^43^ can be used to investigate the structural effect of indel variants. Finally, the study was subject to ascertainment bias, which can be minimized in the future by withholding information obtained by the clinical scientist (ie, variant pathogenicity) from the computational biologist (ie, protein structure analysis).

In conclusion, a homology model of Ca_v_1.4α1 protein with a 64% sequence identity has been used to distinguish all disease-correlated variants from putatively benign, small *CACNA1F* indel variants. Structural analysis can be used to provide clinicians with functional predictions as well as the reasons for the predictions. This approach can be integrated into the ACMG guidelines by diagnostic laboratories to help make informed decisions on patients suspected of having CSNB2. The application of this approach to distinguish and predict indel variants in other rare diseases could further provide valuable information on its accuracy and robustness.

## Supplemental Data

Supplemental Figure S1 The Ramachandran plot of the Ca_v_1.4α1 homology model. The dihedral angles, characterizing the protein chain fold, located in **light blue** and **dark blue areas** correspond to favored and allowed residues, respectively, with the outliers located outside of these regions. The plot was produced by using MolProbity^26^ at *http://molprobity.biochem.duke.edu/index.php* (accessed February 18, 2022).

Supplemental Figure S2 Prediction of the disordered regions in the *CACNA1F* sequence using IUPred. Regions with a prediction score higher than 0.5 (highlighted by the **black line**) are predicted to be disordered.
