# Supplementary material for: Assessing the Pathogenicity of In-Frame CACNA1F Indel Variants Using Structural Modeling
Source: J Mol Diagn. 2022 Oct 1;24(12):1232–9. doi: 10.1016/j.jmoldx.2022.09.005 (PMC12179508; doi:10.1016/j.jmoldx.2022.09.005)
Supplement: Supplemental Figure S1 — The Ramachandran plot of the Cav1.4α1 homology model. The dihedral angles, characterizing the protein chain fold, located in light blue and dark blue areas correspond to favored and allowed residues, respectively, with the outliers located outside of these regions. The plot was produced by using MolProbity26 at http://molprobity.biochem.duke.edu/index.php (accessed February 18, 2022). [file mmc1.pdf]

MolProbity Ramachandran analysis

cacna1fmodelH_cleanFH.pdb, model 1

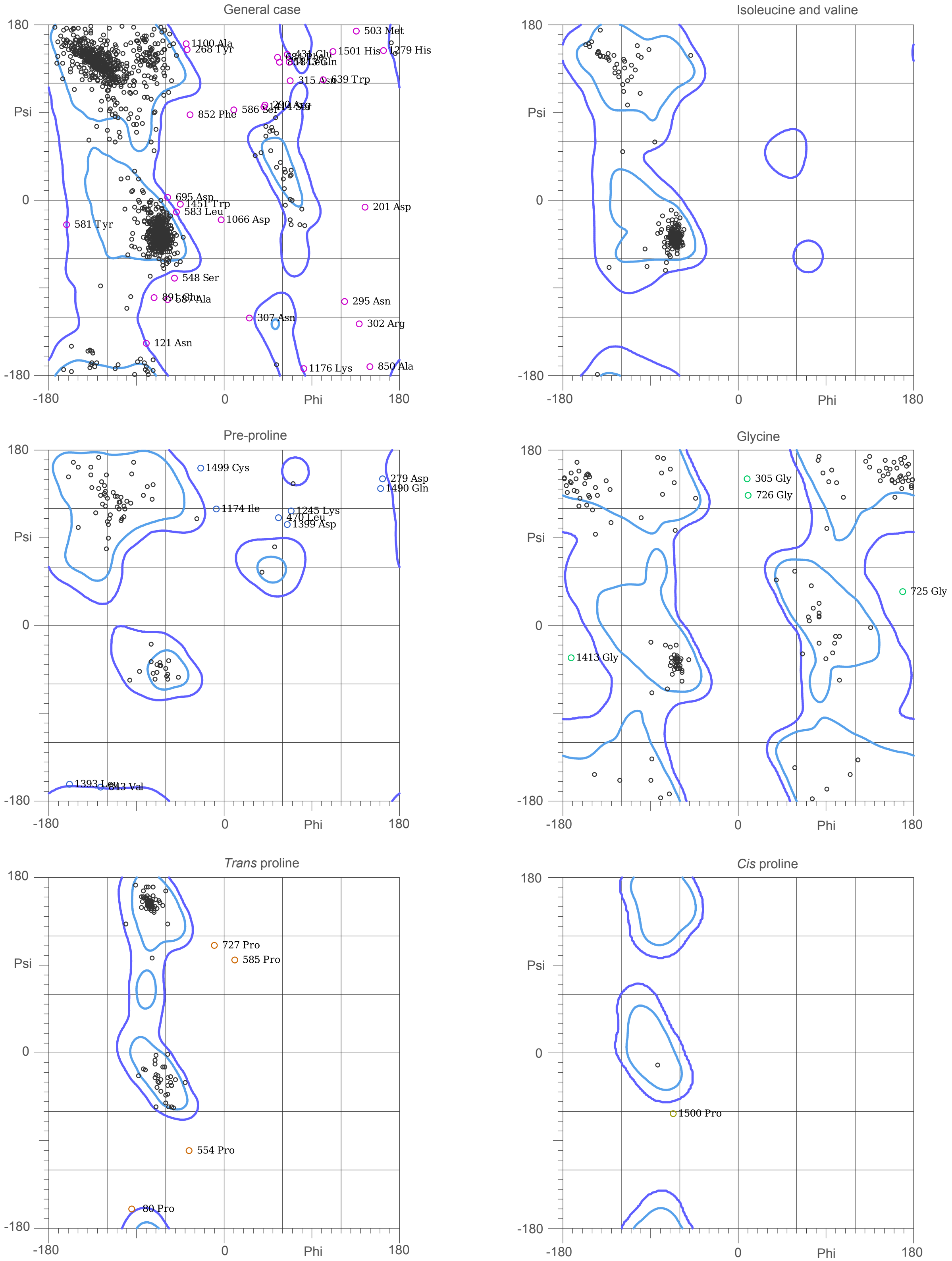

92.7% (1831/1975) of all residues were in favored (98%) regions.
97.6% (1927/1975) of all residues were in allowed (>99.8%) regions.

There were 48 outliers (phi, psi):
80 Pro (-95.4, -160.5)
121 Asn (-80.1, -147.2)
201 Asp (145.2, -7.7)
268 Tyr (-38.4, 155.2)
279 Asp (163.1, 151.7)
290 Arg (42.9, 98.4)
295 Asn (124.9, -104.8)
302 Arg (139.5, -127.4)
305 Gly (9.2, 151.7)
307 Asn (26.2, -121.2)
315 Asn (68.6, 123.2)
431 Glu (65.2, 150.9)
470 Leu (56.2, 111.9)
503 Met (136.3, 174.4)
548 Ser (-51.3, -80.6)
554 Pro (-36.4, -100.1)
581 Tyr (-162.2, -25.1)
583 Leu (-49.2, -12.4)
585 Pro (11.4, 96.8)
586 Ser (10.8, 93.6)
587 Ala (-58.6, -102.5)
639 Trp (102.3, 124.3)
684 Phe (55.4, 147.7)
695 Asp (-58.5, 3.6)
725 Gly (169.5, 36.0)
726 Gly (10.9, 134.6)
727 Pro (-10.2, 111.6)
843 Val (-128.0, -166.3)
850 Ala (150.9, -171.9)
852 Phe (-35.9, 88.1)
854 Leu (57.5, 142.6)
891 Glu (-72.9, -100.5)
1066 Asp (-3.9, -20.4)
1100 Ala (-39.5, 161.8)
1174 Ile (-8.8, 120.9)
1176 Lys (82.5, -173.8)
1245 Lys (69.7, 118.1)
1279 His (164.8, 154.2)
1393 Leu (-159.3, -163.9)
1399 Asp (65.4, 104.9)
1413 Gly (-173.0, -33.8)
1414 Ser (41.7, 97.0)
1451 Trp (-45.5, -4.6)
1490 Gln (161.3, 141.9)
1499 Cys (-24.5, 162.3)
1500 Pro (-67.2, -62.8)
1501 His (112.8, 153.2)
1843 Gln (67.9, 142.7)
