# Supplementary figures and images for: Assessing the Pathogenicity of In-Frame CACNA1F Indel Variants Using Structural Modeling
Source: J Mol Diagn. 2022 Oct 1;24(12):1232–9. doi: 10.1016/j.jmoldx.2022.09.005 (PMC12179508; doi:10.1016/j.jmoldx.2022.09.005)

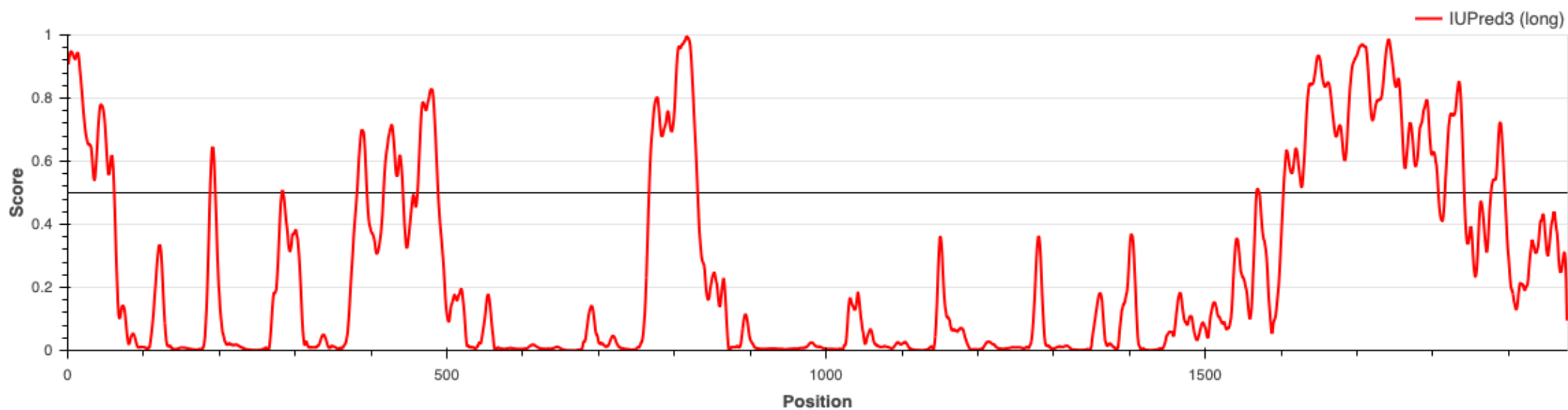

Supplement: Supplemental Figure S2 — Prediction of the disordered regions in the CACNA1F sequence using IUPred. Regions with a prediction score higher than 0.5 (highlighted by the black line) are predicted to be disordered. [file mmc2.pdf]
