# Supplementary material for: Assessing the Pathogenicity of In-Frame CACNA1F Indel Variants Using Structural Modeling
Source: J Mol Diagn. 2022 Oct 1;24(12):1232–9. doi: 10.1016/j.jmoldx.2022.09.005 (PMC12179508; doi:10.1016/j.jmoldx.2022.09.005)
Supplement: Supplemental Table S1 [file mmc3.docx]

Table S1. In-frame CACNA1F insertion and/or deletion (indel) variants were classified using the American College of Medical Genetics and Genomics and Association of Molecular Pathology (ACMG) guidelines [^20^](#_ENREF_20). The variants were identified in individuals with CSNB2 (dataset A) or in individuals who either did not have CSNB2 or who had CSNB2 but had a previously-identified pathogenic variant (dataset B). The variants denoted with an asterisk were found in a low complexity region.

| Variant | ACMG Classification/Criteria | Notes |
| --- | --- | --- |
| ***Dataset A*** |  |  |
| c.466_469delinsGTAGGGGTGCTCCACCCCGTAGGGGAGCTCCACC p.(Ser156_Ala157delins ValGlyValLeuHisProValGlyValLeuHisPro) | LP (PM2, PM4, PS4_M) | PM2 - absent on gnomAD. delins - *p.(Ser156_Ala157delins12)* (in-frame). PM4 - Protein length changes as a result of in-frame deletions/insertions in a nonrepeat region or stop-loss variants (moderate level). Not seen before in-house. Identified in one patient in Nakamura et al (2001) and Boycott (2001) - PS4_M from 2 papers [^32^](#_ENREF_32)^,35^. |
| c.495_496insTACCTA p.(Leu165_Leu166insTyrLeu) | VUS (PM2, PM4, PS4_P) | PM2 - absent on gnomAD. PM4 - Protein length changes as a result of in-frame deletions/insertions in a nonrepeat region or stop-loss variants (moderate level). Not seen before in-house. Identified in 1 patient [^33^](#_ENREF_33) - PS4_P. |
| c.952_954delTTC p.(Phe318del) | VUS (PM2, PS4_M) | PM2 - absent on gnomAD. Within a run of 3 phenylalnines. Not seen before in-house. Seen in one patient in Bijveld (2013) ^36^. Identified in Boycott (2001) [^32^](#_ENREF_32) though does not seem to specify the number of affected individuals. Applied PS4 (moderate level) as a minimum as not a singleton and also have patient from Bijveld paper. Also listed in Zeitz (2019) [^33^](#_ENREF_33). |
| c.1004_1009delTGCTCT p.(Val335_Tyr337delinsAsp) | VUS (PM2, PM4, PS4_P) | PM2 - absent on gnomAD. PM4 - Protein length changes as a result of in-frame deletions/insertions in a nonrepeat region or stop-loss variants (moderate level). Not seen before in-house. Listed in Zeitz (2015) [^3^](#_ENREF_3) though no details given regarding number of affected individuals in paper. Seen in one case - PS4 supporting. |
| c.1612_1632delCTCGTCTTCCTCAACACGTTG p.(Leu538_Leu544del) | VUS (PM2, PS4_M, PP4_P) | PM2 - absent on gnomAD, PM4 not applied as run of 3 leucines. Seen twice in-house (18005766 [congenital nystagmus, photophobia] & 18006522 [?CSNB]). Applied PS4 moderate assuming this finding fits with both patients clinical presentation [variant has been previously identified in multiple (two or more) apparently unrelated affected individuals and has not been reported in gnomAD]. |
| c.2829_2830delGGinsCT p.(Leu943_Asp944delinsPheTyr) | VUS (PM2, PM4, PS4_P) | PM2 - absent on gnomAD. PM4 - Protein length changes as a result of in-frame deletions/insertions in a nonrepeat region or stop-loss variants (moderate level). Not seen before in-house. Listed in Zeitz (2015)^3^ (reported as c.2796_2797delinsCT p.Leu932_Asp933delinsPheTyr). Identified in a single case - PS4_P. |
| c.3009_3011delCAT p.(Ile1003del) | VUS (PM2, PM4_P, PS4_M) | PM2 - absent on gnomAD. PM4_P - Protein length changes as a result of in-frame deletions/insertions in a nonrepeat region or stop-loss variants (supporting level as a single amino acid). Not seen before in-house. Listed in Boycott (2001) [^32^](#_ENREF_32) and in Zeitz et al (2019) [^33^](#_ENREF_33) - PS4_M. |
| c.3658_3669delGTCCATGGCATA p.(Tyr1220_Asp1223del) | VUS (PM2, PM4, PS4_P) | PM2 - absent on gnomAD. PM4 - Protein length changes as a result of in-frame deletions/insertions in a nonrepeat region or stop-loss variants (moderate level). Not seen before in-house. Identified in Wutz et al (2002) [^31^](#_ENREF_31), no details given regarding the number of affected individuals. Assume as a minimum there is 1 affected individual - apply PS4_P. |
| c.3691_3702delAGTGAAGAGGCC p.(Gly1231_Thr1234del) | VUS (PM2, PM4, PS4_P) | PM2 - absent on gnomAD. PM4 - Protein length changes as a result of in-frame deletions/insertions in a nonrepeat region or stop-loss variants (moderate level). Not seen before in-house. Identified in Strom (1998) [^34^](#_ENREF_34) reported as del3658-3669 30 12-bp deletion. No details of affected individuals - assume singleton - PS4_P. |
| c.4093_4095delAAC p.(Asn1365del) | VUS (PM2, PS4_P, PP4_P) | PM2 - absent on gnomAD, PM4 - not applied as within a run of 3 AACs [Protein length changes as a result of in-frame deletions/insertions in a nonrepeat region or stop-loss variants, to be applied at a supporting level if a deletion of a single amino acid]. Seen once in-house. |
|  |  |  |
| ***Dataset B*** |  |  |
| c.1466_1468delAGG p.(Glu489del) | VUS (BS1) | gnomAD 3/141173 1 hemizygote 0.002125%, within a run of AGGs, no evidence in the literature. BS1_S - Allele frequency is greater than expected for disorder. |
| c.5195_5197delAAG p.(Glu1732del) | VUS (BS1) | gnomAD 3/186240 1 hemizygote 0.001611%. BS1_S - Allele frequency is greater than expected for disorder. |
| c.5866_5868delGAG p.(Glu1956del) | VUS (BS1) | gnomAD 2/182111 1 hemizygote 0.001098%. BS1_S - Allele frequency is greater than expected for disorder. |
| c.2442_2444delGGA p.(Glu825del) * | VUS (BS1) | gnomAD 76/141706 6 hemizygotes 0.05363%. BS1_S - Allele frequency is greater than expected for disorder. |
| c.2457_2474dupAGAGGAAGAAGAGGAAGA p.(Glu820_Glu825dup) * | VUS (BS1) | gnomAD 11/147711 8 hemizygotes 0.007447%. BS1_S - Allele frequency is greater than expected for disorder. |
| c.2466_2474delAGAGGAAGA p.(Glu823_Glu825del) * | VUS (BS1) | gnomAD 118/168592 26 hemizygote 0.06999%. BS1_S - Allele frequency is greater than expected for disorder. Poly Glu track. |
| c.2445_2450delAGAAGA p.(Glu824_Glu825del) * | VUS (BS1) | gnomAD 1/130487 1 hemizygote 0.0007664%. BS1_S - Allele frequency is greater than expected for disorder. |
| c.2466_2474dupAGAGGAAGA p.(Glu823_Glu825dup) * | VUS (BS1) | gnomAD: 99/168592 30 hemizygotes 0.05872%. BS1_S - Allele frequency is greater than expected for disorder. |
| c.2439_2444dupGGAGGA p.(Glu824_Glu825dup) * | VUS (BS1) | gnomAD: 4/120887 1 hemizygote 0.003309%. BS1_S - Allele frequency is greater than expected for disorder. |
| c.2442_2444dupGGA p.(Glu825dup) * | VUS (BS1) | gnomAD: 5575/141706 341 homozygotes 1128 hemizygotes 3.934%. BS1_S - Allele frequency is greater than expected for disorder. |
