# Supplementary material for: Assessing the Pathogenicity of In-Frame CACNA1F Indel Variants Using Structural Modeling
Source: J Mol Diagn. 2022 Oct 1;24(12):1232–9. doi: 10.1016/j.jmoldx.2022.09.005 (PMC12179508; doi:10.1016/j.jmoldx.2022.09.005)
Supplement: Supplemental Table S2 [file mmc4.docx]

Supplemental Table S2. The pathogenic/likely pathogenic variants seen with the putatively benign *CACNA1F* variant *c.2442_2444dupGGA p.(Glu825dup)* in eight patients within the in-house Manchester Genomic Diagnostic Laboratory (MGDL) database ; hom: homozygous; het: heterozygous.

| Sex | Age | Benign variant identified | Clinical diagnosis | Molecular diagnosis | ACMG classification |
| --- | --- | --- | --- | --- | --- |
| Female | 32 | *CACNA1F c.2445_2444dupGGA p.Glu825dup* | Usher syndrome | *USH2A c.2299delG* het  *USH2A c.949C>A p.(Arg317Arg)* het | Both are likely pathogenic |
| Male | 20 | *CACNA1F c.2445_2444dupGGA p.Glu825dup* | Usher syndrome | *CDH23 c.2289+1G>A* het  *CDH exon2-11 deletion* het | Both are likely pathogenic |
| Female | 8 | *CACNA1F c.2445_2444dupGGA p.Glu825dup* | Leber Congenital Amaurosis | *WDR19 c.3533 G>A p.(Arg1178Gln)* hom | Likely pathogenic |
| Female | 35 | *CACNA1F c.2445_2444dupGGA p.Glu825dup* | Cone dystrophy | - | - |
| Female | 29 | *CACNA1F c.2445_2444dupGGA p.Glu825dup* | Rod-Cone dystrophy | *RHO c.512C>A p.(Pro171Gln)* het | Likely pathogenic |
| Female | 13 | *CACNA1F c.2445_2444dupGGA p.Glu825dup* | Fundus albipunctatus | *RLBP1 c.701G?A p.(Arg234Gln)* hom | Likely pathogenic |
| Female | 79 | *CACNA1F c.2445_2444dupGGA p.Glu825dup* | AR retinitis pigmentosa | *PDE6B c.1798G>A p.(Asp600Asn)* hom | Likely pathogenic |
| Male | 41 | *CACNA1F c.2445_2444dupGGA p.Glu825dup* | Reduced night and colour vision | *EYSc.6646T>C p.(Cys2216Arg)* hom | VUS |
