# Supplementary material for: Assessing the Pathogenicity of In-Frame CACNA1F Indel Variants Using Structural Modeling
Source: J Mol Diagn. 2022 Oct 1;24(12):1232–9. doi: 10.1016/j.jmoldx.2022.09.005 (PMC12179508; doi:10.1016/j.jmoldx.2022.09.005)
Supplement: Supplemental Table S3 [file mmc5.docx]

Supplemental Table S3. The sequence alignment of the template structure (rabbit Ca_v_1.1 complex; PDB ID: 5GJV) and human *CACNA1F* using Clustal Omega v1.2.4. Identical residues are marked with ’*’, conserved and semi-conserved substitutions are marked with ‘:’ and ‘.’, respectively, and dissimilar substitutions or gaps are unmarked.

sp|O60840|CAC1F_HUMAN MSESEGGKDTTPEPSPANGAGPGPEWGLCPGPPAVEGESSGASGLGTPKRRNQHSKHKTV 60

5GJV_1|Chain ---------------------------MEPSSPQDE-------GLRK-------KQPKKP 19

: *. * * ** . .: *.

sp|O60840|CAC1F_HUMAN AVASAQRSPRALFCLTLANPLRRSCISIVEWKPFDILILLTIFANCVALGVYIPFPEDDS 120

5GJV_1|Chain LPEVLPRPPRALFCLTLQNPLRKACISIVEWKPFETIILLTIFANCVALAVYLPMPEDDN 79

* ********* ****::**********: :************.**:*:****.

sp|O60840|CAC1F_HUMAN NTANHNLEQVEYVFLVIFTVETVLKIVAYGLVLHPSAYIRNGWNLLDFIIVVVGLFSVLL 180

5GJV_1|Chain NSLNLGLEKLEYFFLTVFSIEAAMKIIAYGFLFHQDAYLRSGWNVLDFIIVFLGVFTAIL 139

*: * .**::**.**.:*::*:.:**:***:::* .**:*.***:******.:*:*:.:*

sp|O60840|CAC1F_HUMAN EQGPGRPGDAPHTGGKPGGFDVKALRAFRVLRPLRLVSGVPSLHIVLNSIMKALVPLLHI 240

5GJV_1|Chain EQVNVIQSNTAPMSSKGAGLDVKALRAFRVLRPLRLVSGVPSLQVVLNSIFKAMLPLFHI 199

** .:: ..* .*:***********************::*****:**::**:**

sp|O60840|CAC1F_HUMAN ALLVLFVIIIYAIIGLELFLGRMHKTCYFLGSDMEA---EEDPSPCASSGSGRACTLNQT 297

5GJV_1|Chain ALLVLFMVIIYAIIGLELFKGKMHKTCYYIGTDIVATVENEKPSPCARTGSGRPCTINGS 259

******::*********** *:******::*:*: * :*.***** :**** **:* :

sp|O60840|CAC1F_HUMAN ECRGRWPGPNGGITNFDNFFFAMLTVFQCVTMEGWTDVLYWMQDAMGYELPWVYFVSLVI 357

5GJV_1|Chain ECRGGWPGPNHGITHFDNFGFSMLTVYQCITMEGWTDVLYWVNDAIGNEWPWIYFVTLIL 319

**** ***** ***:**** *:****:**:***********::**:* * **:***:*::

sp|O60840|CAC1F_HUMAN FGSFFVLNLVLGVLSGEFSKEREKAKARGDFQKQREKQQMEEDLRGYLDWITQAEELDME 417

5GJV_1|Chain LGSFFILNLVLGVLSGEFTKEREKAKSRGTFQKLREKQQLEEDLRGYMSWITQGEVMDVE 379

:****:************:*******:** *** *****:*******:.****.* :*:*

sp|O60840|CAC1F_HUMAN DPSADDNLGSMAEEGRAGHRPQLAELTNRRRGRLRWFSHSTRSTHSTSSHASLPASDTGS 477

5GJV_1|Chain DLREGKL--SLEE-G---------------------------------------GSDTES 397

* .. *: * * .*** *

sp|O60840|CAC1F_HUMAN MTETQGDEDEEEGALASCTRCLNKIMKTRVCRRLRRANRVLRARCRRAVKSNACYWAVLL 537

5GJV_1|Chain LYEI---------------EGLNKI--IQFIRHWRQWNRVFRWKCHDLVKSRVFYWLVIL 440

: * . **** :. *: *: ***:* :*: ***.. ** *:*

sp|O60840|CAC1F_HUMAN LVFLNTLTIASEHHGQPVWLTQIQEYANKVLLCLFTVEMLLKLYGLGPSAYVSSFFNRFD 597

5GJV_1|Chain IVALNTLSIASEHHNQPLWLTHLQDIANRVLLSLFTIEMLLKMYGLGLRQYFMSIFNRFD 500

:* ****:******.**:***::*: **:***.***:*****:**** *. *:*****

sp|O60840|CAC1F_HUMAN CFVVCGGILETTLVEVGAMQPLGISVLRCVRLLRIFKVTRHWASLSNLVASLLNSMKSIA 657

5GJV_1|Chain CFVVCSGILELLLVESGAMTPLGISVLRCIRLLRLFKITKYWTSLSNLVASLLNSIRSIA 560

*****.**** *** *** *********:****:**:*::*:************::***

sp|O60840|CAC1F_HUMAN SLLLLLFLFIIIFSLLGMQLFGGKFNFDQTHTKRSTFDTFPQALLTVFQILTGEDWNVVM 717

5GJV_1|Chain SLLLLLFLFIIIFALLGMQLFGGRYDFEDTEVRRSNFDNFPQALISVFQVLTGEDWNSVM 620

*************:*********:::*::*..:**.**.*****::***:******* **

sp|O60840|CAC1F_HUMAN YDGIMAYGGPFFPGMLVCIYFIILFICGNYILLNVFLAIAVDNLASGDAGTAKDKGGEKS 777

5GJV_1|Chain YNGIMAYGGPSYPGVLVCIYFIILFVCGNYILLNVFLAIAVDNLAEAESLTSAQKAKAEE 680

*:******** :**:**********:*******************..:: *: :*. :.

sp|O60840|CAC1F_HUMAN -----NEKDLPQENEGLVPGVEKEEEEGARREGADME----EE----------------E 812

5GJV_1|Chain RKRRKMSRGLPDKTEEEKSVMAKKLEQKPKGEGIPTTAKLKVDEFESNVNEVKDPYPSAD 740

.:.**::.* : *: *: : ** : :

sp|O60840|CAC1F_HUMAN EEEEEEEEEEEEEGAGGVELLQEVVPKEKVVPIPEGSAFFCLSQTNPLRKGCHTLIHHHV 872

5GJV_1|Chain FPGDDEEDEPEIPVSPRPRPLAELQLKEKAVPIPEASSFFIFSPTNKVRVLCHRIVNATW 800

::**:* * : . * *: ***.*****.*:** :* ** :* ** :::

sp|O60840|CAC1F_HUMAN FTNLILVFIILSSVSLAAEDPIRAHSFRNHILGYFDYAFTSIFTVEILLKMTVFGAFLHR 932

5GJV_1|Chain FTNFILLFILLSSAALAAEDPIRAESVRNQILGYFDIAFTSVFTVEIVLKMTTYGAFLHK 860

***:**:**:***.:*********.*.**:****** ****:*****:****.:*****:

sp|O60840|CAC1F_HUMAN GSFCRSWFNMLDLLVVSVSLISFGIHSSAISVVKILRVLRVLRPLRAINRAKGLKHVVQC 992

5GJV_1|Chain GSFCRNYFNILDLLVVAVSLISMGLESSTISVVKILRVLRVLRPLRAINRAKGLKHVVQC 920

*****.:**:******:*****:*:.**:*******************************

sp|O60840|CAC1F_HUMAN VFVAIRTIGNIMIVTTLLQFMFACIGVQLFKGKFYTCTDEAKHTPQECKGSFLVYPDGDV 1052

5GJV_1|Chain VFVAIRTIGNIVLVTTLLQFMFACIGVQLFKGKFFSCNDLSKMTEEECRGYYYVYKDGDP 980

***********::*********************::*.* :* * :**:* : ** ***

sp|O60840|CAC1F_HUMAN SRPLVRERLWVNSDFNFDNVLSAMMALFTVSTFEGWPALLYKAIDAYAEDHGPIYNYRVE 1112

5GJV_1|Chain TQMELRPRQWIHNDFHFDNVLSAMMSLFTVSTFEGWPQLLYRAIDSNEEDMGPVYNNRVE 1040

:: :* * *::.**:*********:*********** ***:***: ** **:** ***

sp|O60840|CAC1F_HUMAN ISVFFIVYIIIIAFFMMNIFVGFVIITFRAQGEQEYQNCELDKNQRQCVEYALKAQPLRR 1172

5GJV_1|Chain MAIFFIIYIILIAFFMMNIFVGFVIVTFQEQGETEYKNCELDKNQRQCVQYALKARPLRC 1100

:::***:***:**************:**: *** **:************:*****:***

sp|O60840|CAC1F_HUMAN YIPKNPHQYRVWATVNSAAFEYLMFLLILLNTVALAMQHYEQTAPFNYAMDILNMVFTGL 1232

5GJV_1|Chain YIPKNPYQYQVWYVVTSSYFEYLMFALIMLNTICLGMQHYHQSEEMNHISDILNVAFTII 1160

******:**:** .*.*: ****** **:***:.*.****.*: :*: ****:.** :

sp|O60840|CAC1F_HUMAN FTIEMVLKIIAFKPKHYFTDAWNTFDALIVVGSIVDIAVTEVNN------------GGHL 1280

5GJV_1|Chain FTLEMILKLLAFKARGYFGDPWNVFDFLIVIGSIIDVILSEIDTFLASSGGLYCLGGGCG 1220

**:**:**::*** : ** * **.** ***:***:*: ::*::. **

sp|O60840|CAC1F_HUMAN GESSEDSSRISITFFRLFRVMRLVKLLSKGEGIRTLLWTFIKSFQALPYVALLIAMIFFI 1340

5GJV_1|Chain NVDPDESARISSAFFRLFRVMRLIKLLSRAEGVRTLLWTFIKSFQALPYVALLIVMLFFI 1280

. . ::*:*** :**********:****:.**:*********************.*:***

sp|O60840|CAC1F_HUMAN YAVIGMQMFGKVALQDGTQINRNNNFQTFPQAVLLLFRCATGEAWQEIMLASLPGNRCDP 1400

5GJV_1|Chain YAVIGMQMFGKIALVDGTQINRNNNFQTFPQAVLLLFRCATGEAWQEILLACSYGKLCDP 1340

***********:** *********************************:**. *: ***

sp|O60840|CAC1F_HUMAN ESDFGPGEEFTCGSNFAIAYFISFFMLCAFLIINLFVAVIMDNFDYLTRDWSILGPHHLD 1460

5GJV_1|Chain ESDYAPGEEYTCGTNFAYYYFISFYMLCAFLIINLFVAVIMDNFDYLTRDWSILGPHHLD 1400

***:.****:***:*** *****:***********************************

sp|O60840|CAC1F_HUMAN EFKRIWSEYDPGAKGRIKHLDVVALLRRIQPPLGFGKLCPHRVACKRLVAMNMPLNSDGT 1520

5GJV_1|Chain EFKAIWAEYDPEAKGRIKHLDVVTLLRRIQPPLGFGKFCPHRVACKRLVGMNMPLNSDGT 1460

*** **:**** ***********:*************:***********.**********

sp|O60840|CAC1F_HUMAN VTFNATLFALVRTSLKIKTEGNLEQANQELRIVIKKIWKRMKQKLLDEVIPPPDEEEVTV 1580

5GJV_1|Chain VTFNATLFALVRTALKIKTEGNFEQANEELRAIIKKIWKRTSMKLLDQVIPPIGDDEVTV 1520

*************:********:****:*** :******* . ****:**** .::****

sp|O60840|CAC1F_HUMAN GKFYATFLIQDYFRKFRRRKEKGLLGNDAAPSTSSALQAGLRSLQD-LGPEMRQALTCDT 1639

5GJV_1|Chain GKFYATFLIQEHFRKFMKRQEEYY-GYR-PKKDTVQIQAGLRTIEEEAAPEIRRTISGDL 1578

**********::**** :*:*: * . : :*****:::: .**:*:::: *

sp|O60840|CAC1F_HUMAN EEEEEEGQEGVEEEDEKDLETNKATMVSQPSARRGSGISVSLPVGDRLPDSLSFGPSDDD 1699

5GJV_1|Chain TAEEELERAMVEAAMEERIFRRTGGLFGQVDTFLER--TNSLPPVMANQRPLQFAEIEM- 1635

*** : ** *: : ... :..* .: : *** *.*. :

sp|O60840|CAC1F_HUMAN RGTPTSSQPSVPQAGSNTHRRGSGALIFTIPEEGNSQPKGTKGQNKQDEDEEVPDRLSYL 1759

5GJV_1|Chain ------------------EELESPVFLEDFPQDARTNPLARANTNNANANVAYGNSNHSN 1677

.. * .:: :*::..::* . . *: : : :

sp|O60840|CAC1F_HUMAN DEQAGTPPCSVLLPPHRAQ-RYMDGHLVPRRRLLPPTPAGRKPSFTIQCLQRQGSCEDLP 1818

5GJV_1|Chain NQMFSSVHCEREFPGEAETPAAGRGALSHSHRALGPHSK--------PCAGKLN--GQLV 1727

:: .: *. :* . * * :* * * * : . :*

sp|O60840|CAC1F_HUMAN IPGTYHRGRNSGPNRAQGSWATPPQRGRLLYAPLLLVEEGAAGEGYLGRSSGPLRTFTCL 1878

5GJV_1|Chain QPGMPIN--QAPPAPCQQPSTDPPERGQRRTSLT----------GSLQD-EAPQRR---- 1770

** . :: * .* : **:**: : * * ..* *

sp|O60840|CAC1F_HUMAN HVPGTHSDPSHGKRGSADSLVEAVLISEGLGLFARDPRFVALAKQEIADACRLTLDEMDN 1938

5GJV_1|Chain --SSEGSTPR-RPAPATALLIQEALVRGGLDTLAADAGFVTATSQALADACQMEPEEVEV 1827

. * * :: *:: .*: **. :* * **: :.* :****:: :*::

sp|O60840|CAC1F_HUMAN AASDLLAQGTSSLY--SDEESILSRFDEEDLGDEMACVHAL----- 1977

5GJV_1|Chain AATELLKARESVQGMASVPGSLSRRSSLGSLDQVQGSQETLIPPRP 1873

**::** * * *: * . .*.: .. .:*
