# Supplementary material for: Assessing the Pathogenicity of In-Frame CACNA1F Indel Variants Using Structural Modeling
Source: J Mol Diagn. 2022 Oct 1;24(12):1232–9. doi: 10.1016/j.jmoldx.2022.09.005 (PMC12179508; doi:10.1016/j.jmoldx.2022.09.005)
Supplement: Supplemental Table S4 [file mmc6.docx]

Supplemental Table S4. The structural analysis of the eight disease-correlated small in-frame *CACNA1F* indel variants. The analyses were carried out using a homologous model of Ca_v_1.4α1 (ENST00000376265.2).

| *c.466-469delAGCGinsGTAGGGGTGCTCCACCCCGTAGGGGAGCTCCACC p.(Ser156_Ala157delins ValGlyValLeuHisProValGlyValLeuHisPro)* |
| --- |
| This variant is on a transmembrane region bordering the intracellular region. The replacing of two residues small in size and non-hydrophobic with a repeat of 12 residues, including six hydrophobic, is likely to have a functional effect on the protein. These residues are added to a short loop between the two α-helices likely exposed in the intracellular environment which could lead to structural destabilization. |
| *c.495_496insTACCTA p.(Leu165_Leu166insTyrLeu)* |
| This variant is in the middle of a transmembrane helix. Considering the 3.6 residues in a turn in a helix, the insertion of these two residues shifts the positions of the other residues in the helix which can lead to the disruption of hydrophobic interactions formed between the residues of this helix and those of the neighboring helices. This disruption could result in an ineffective voltage-gated domain and a destabilized protein. |
| *c.952_954delTTC p.(Phe318del)* |
| This variant is in the first turn of a short helix in a region involved in calcium ion intake. The deletion of Phe318 could lead to the displacement of the ensuing residues and disturb the structure of the helix. Also, the deletion of Phe318 removes the hydrogen bonds it forms with Asn312 which could be destabilizing. |
| *c.1004_1009delTGCTCT p.(Val335_Tyr337delinsAsp)* |
| This variant is in one of the short helices in a region involved in calcium ion intake. The deletion of three hydrophobic residues and the insertion of a charged hydrophilic residue is likely to disturb hydrophobic interactions and likely destabilize the protein by rendering the helix ineffective and the protein dysfunctional. |
| *c.2829_2830delGGinsCT p.(Leu943_Asp944delinsPheTyr)* |
| This variant is in the middle of a helix in the transmembrane region. The replacement of the relatively smaller Leu943 with a larger aromatic Phe is likely to result in van der Waals clashes affecting protein stability. The replacement of the charged Asp944 with the hydrophobic and bulky Tyr is likely to result in the loss of ionic interaction and possible protein destabilization. |
| *c.3009_3011delCAT p.(Ile1003del)* |
| This variant is in a hydrophobic region on a long helix involved in opening the channel by linking the voltage sensing domain to the pore. The deletion of Ile1003 is likely to displace the ensuing residues on this long helix and disrupt hydrophobic interactions resulting in structural destabilization. |
| *c.3658delGTCCATGGCATA p.(Tyr1220_Asp1223del)* |
| This variant is in a transmembrane helix. The deletion of four residues is likely to affect the function of the voltage-gated domain and render the protein dysfunctional. The variant includes the deletion of Asp1223 which is in close proximity to another Asp on an adjacent helix. The deletion of the Asp could affect packing interactions and destabilize the protein. Also, Met1222 forms hydrogen bonds with Gln1210 removal of which could be deleterious. |
| *c.3691_3702delAGTGAAGAGGCC p.(Gly1231_Thr1234del)* |
| This variant is in the middle of a transmembrane helix. The deletion of four residues is likely to affect the existing hydrophobic interactions between the adjacent helices rendering this voltage-gated domain ineffective and the protein dysfunctional. In addition, the deletion of Phe1233 and Thr1234 removes the hydrogen bonds they form with Lys1305 on the adjacent α-helix and Thr1230, respectively, which could be destabilizing. |
